# Cross-Software Radiomic Feature Robustness Assessed by Hierarchical Clustering and Composite Index Analysis: A Multi-Cancer Study on Colorectal and Liver Lesions

**DOI:** 10.3390/bioengineering12121282

**Published:** 2025-11-21

**Authors:** Roberta Fusco, Giulia Festa, Mario Sansone, Sergio Venanzio Setola, Antonio Avallone, Francesco Izzo, Antonella Petrillo, Vincenza Granata

**Affiliations:** 1 Division of Radiology, Istituto Nazionale Tumori IRCCS Fondazione Pascale—IRCCS di Napoli, 80131 Naples, Italy; r.fusco@istitutotumori.na.it (R.F.); s.setola@istitutotumori.na.it (S.V.S.); a.petrillo@istitutotumori.na.it (A.P.); 2 Biomedical Engineering Faculty, Università degli Studi di Napoli Federico II, 80125 Naples, Italy; giul.festa@studenti.unina.it (G.F.);; 3 Clinical Sperimental Abdominal Oncology Unit, Istituto Nazionale Tumori IRCCS Fondazione Pascale—IRCCS di Napoli, 80131 Naples, Italy; a.avallone@istitutotumori.na.it; 4 Division of Epatobiliary Surgical Oncology, Istituto Nazionale Tumori IRCCS Fondazione Pascale—IRCCS di Napoli, 80131 Naples, Italy; f.izzo@istitutotumori.na.it

**Keywords:** radiomics, feature robustness, hierarchical clustering, colorectal cancer, liver metastases, hepatocellular carcinoma

## Abstract

**Background:** Radiomic feature robustness is a key prerequisite for the reproducibility and clinical translation of imaging biomarkers. Variability across software platforms can significantly affect feature consistency, compromising predictive modeling reliability. This study aimed to develop and validate a hierarchical clustering-based workflow for evaluating radiomic feature robustness within and across software platforms, identifying stable and reproducible features suitable for clinical applications. **Methods:** A multi-cancer CT dataset including 97 lesions from 71 patients, comprising primary colorectal cancer (CRC), colorectal liver metastases, and hepatocellular carcinoma (HCC), was analyzed. Radiomic features were extracted using two IBSI-compliant platforms (MM Radiomics of syngo.via Frontier and 3D Slicer with PyRadiomics). Intra-software reliability was assessed through the intraclass correlation coefficient ICC(A,1), while cross-software stability was evaluated using hierarchical clustering validated by the Adjusted Rand Index (ARI). A Composite Index (CI) integrating correlation, distributional similarity, and mean fractional ratio quantified inter-platform feature robustness. **Results:** Over 95% of radiomic features demonstrated good-to-excellent intra-software reliability. Several clustering configurations achieved ARI = 1.0, confirming strong cross-platform concordance. The most robust and recurrent features were predominantly wavelet-derived descriptors and first-order statistics, particularly cluster shade (GLCM-based) and mean intensity-related features. **Conclusions:** The proposed multi-stage framework effectively identifies stable, non-redundant, and transferable radiomic features across IBSI-compliant software platforms. These findings provide a methodological foundation for cross-platform harmonization and enhance the reproducibility of radiomic biomarkers in oncologic imaging.

## 1. Introduction

Radiomics is an emerging field in medical imaging that enables the extraction of a large number of quantitative features from routinely acquired scans, including computed tomography (CT), magnetic resonance imaging (MRI), and positron emission tomography (PET) [1,2,3,4,5,6,7,8,9,10,11,12,13,14]. These features provide a detailed quantitative characterization of tumor phenotypes, capturing subtle aspects of tumor heterogeneity that are often imperceptible to visual assessment. The potential of radiomics lies in its ability to serve as a non-invasive imaging biomarker for diagnosis, prognosis, and prediction of treatment response across multiple cancer types, including colorectal cancer (CRC) and hepatocellular carcinoma (HCC) [1,2,3,4,5,6,7,8,9,10,11,12,13,14,15,16].

Colorectal cancer represents a major global health challenge, with a high incidence and mortality rate. Liver metastases, which develop in a large proportion of CRC patients, constitute a critical prognostic factor. Similarly, HCC is the most prevalent primary liver malignancy. Accurate lesion characterization is therefore essential for optimal patient management and treatment planning. While traditional diagnostic approaches often rely on invasive procedures, radiomics provides a complementary, non-invasive alternative that extracts quantitative tumor information directly from standard clinical images [15,16]. Several studies have demonstrated promising associations between radiomic texture features, particularly those derived from portal venous phase CT, and histopathological grading, suggesting their potential as imaging biomarkers for tumor differentiation [4,11,14,17,18,19].

Despite these advances, the clinical translation of radiomics remains limited by issues of robustness and reproducibility. Radiomic features can be highly sensitive to differences in image acquisition parameters, scanner models, reconstruction algorithms, and segmentation methods [19,20,21]. Such variability can lead to inconsistent feature values, reducing the generalizability and reliability of predictive models. The Image Biomarker Standardisation Initiative (IBSI) has emphasized that demonstrating repeatability and reproducibility is a prerequisite for the clinical adoption of radiomic biomarkers [16].

Across the literature referenced in [1,2,3,4,5,6,7,8,9,10,11,12,13,14], five recurring sources of variability limit clinical translation of CT radiomics: (1) acquisition/reconstruction dependence, (2) segmentation-related variability, (3) gray-level preprocessing and filtering choices (e.g., discretization, wavelet settings), (4) inter-software implementation differences, and (5) feature redundancy and model overfitting that undermine external validity [1,2,3,4,5,6,7,8,9,10,11,12,13,14]. In parallel, standardization efforts led by the IBSI formalize feature definitions and convolutional filters and recommend explicit reporting of repeatability/reproducibility as a prerequisite to modeling [15,16].

To address these challenges, several methodological strategies have been proposed to identify stable and reproducible radiomic features. These include correlation-based analyses (e.g., Pearson or concordance correlation coefficients) to assess agreement between repeated measurements or observers [2,6], as well as unsupervised clustering techniques to identify and group highly correlated features, thereby reducing redundancy. Feature selection methods are then applied to isolate the most informative and non-redundant variables, mitigating overfitting and enhancing model interpretability [15]. In predictive modeling, machine learning algorithms such as Elastic Net regression, decision trees, and Random Forests have demonstrated strong performance in handling high-dimensional radiomic data [7,8,9,10].

In line with these recommendations, our study operationalizes a stability-first screen and cross-software validation to derive a compact, vendor-agnostic feature subset suitable for downstream modeling.

Building upon these foundations, the present study proposes a comprehensive workflow to systematically evaluate the robustness of radiomic features, both within a single platform (intra-software) and across different platforms (cross-software). This study focuses on features extracted from primary colorectal cancer (CRC), liver metastases by CRC, and HCC. The proposed workflow integrates correlation analyses, clustering techniques, and advanced feature selection methods to identify stable, reproducible, and generalizable radiomic features, thereby facilitating their use in robust and clinically translatable predictive models.

## 2. Methods

### 2.1. Dataset Characteristics

The local ethics committee approved this study (Deliberation No. 323 of 5 March 2023 of the local Institute). The study population consists of a multicancer dataset comprising primary CRC lesions, liver metastases from CRC and HCC.

We retrospectively screened consecutive patients from our institutional surgical database with histologically confirmed primary CRC, colorectal liver metastases (CRLM), or HCC who had pre-surgical, contrast-enhanced CT available. Inclusion required availability of a diagnostic CT examination performed at our institution including a portal venous phase acquired ~90 s after contrast injection, and at least one target lesion clearly visible and amenable to semi-automatic three-dimensional segmentation. When a patient had multiple eligible lesions, all visible lesions meeting the above criteria were included to reflect real-world imaging heterogeneity and to increase stability assessment across software platforms. We excluded CT examinations with severe motion or beam-hardening artifacts, missing portal venous phase, incomplete or corrupted DICOM metadata and lesions that were too small or poorly conspicuous.

According to the inclusion and exclusion criteria, we analyzed 97 lesions (16 HCC, 45 CRC and 36 liver metastases from CRC) in 71 patients with a median age of 62 years (range 35–80 years).

### 2.2. CT Imaging Protocol

CT imaging was conducted using a 64-slice detector scanner (Optima 660, GE Healthcare, Chicago, IL, USA) or Dual Energy Spectral CT (Somatom Drive Dual Source CT System 256 slice, Siemens Healthineers, Erlangen, Germany).

The scan settings included 80–140 kVp, 100–470 mA, with a 2–2.5 mm section thickness. A non-ionic contrast agent was administered at 3 mL/s using an automatic injector (Empower CTA, EZ-EM Inc., New York, NY, USA). For the analysis, only the portal venous phase, obtained 90 s post-contrast injection, was used.

### 2.3. Image Segmentation and Radiomic Feature Extraction

**Figure 1:**
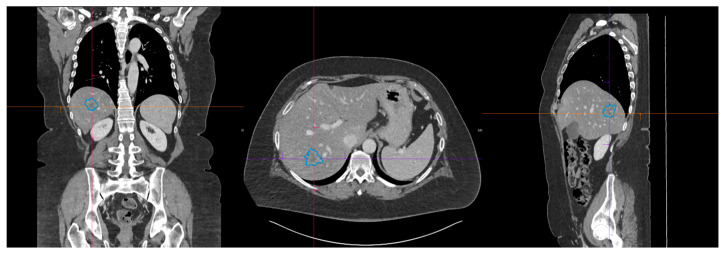
Portal venous phase CT displayed as multiplanar reconstructions: coronal (**left**), axial (**center**), and sagittal (**right**). The blue contour delineates the final 3D region of interest (ROI) of a representative hepatic lesion; contours are projected on each plane for visualization.

Lesion segmentation was performed using the MM Radiomics module within the syngo.via Frontier platform (Siemens Healthineers, Erlangen, Germany). This tool enables semi-automatic three-dimensional segmentation based on artificial intelligence-assisted algorithms (e.g., random walker), allowing the operator to provide an initial seed point or contour, which is then refined automatically (see Figure 1).

All segmentations were subsequently reviewed and, when necessary, manually adjusted in consensus by two expert abdominal radiologists (each with >10 years of experience in oncologic imaging) to ensure anatomical accuracy and consistency across cases. The final binary masks (Region of Interest, ROI) were exported from syngo.via Frontier in DICOM-SEG format and subsequently imported into 3D Slicer (version 5.6.2; www.slicer.org) for radiomic feature extraction.

Radiomic features were extracted using two widely adopted and Image Biomarker Standardisation Initiative (IBSI)-compliant platforms: syngo.via Frontier (Siemens Healthineers; hereafter Platform A) and 3D Slicer (version 5.6.2, with the PyRadiomics module; hereafter Platform B).

When a patient presented multiple eligible lesions, each lesion was segmented independently and treated as a separate observational unit in the lesion-level analysis. Our study is designed to assess cross-software radiomic feature robustness at the lesion level, rather than to draw patient-level inferences.

Platform A—syngo.via Frontier (MM Radiomics Frontier, Siemens Healthineers): syngo.via Frontier is a commercial imaging research platform integrated within the Siemens syngo.via environment and designed for quantitative image analysis. The MM Radiomics Frontier module enables the direct import of DICOM images, semi-automatic 3D segmentation of lesions, and subsequent extraction of radiomic features. The software automatically computes first-order, shape, and texture features using an embedded implementation of the PyRadiomics library, compliant with IBSI. Feature values can be exported in tabular format for downstream statistical and machine-learning analyses.

Platform B—3D Slicer with PyRadiomics extension: 3D Slicer is an open-source, cross-platform software for visualization, segmentation, and quantitative analysis of medical images. Radiomic feature extraction was performed using the SlicerRadiomics extension, which employs the PyRadiomics engine as its computational backend. The software computes a comprehensive set of radiomic features.

Both platforms allow users to extract radiomic features including first-order statistics, shape descriptors, and textural matrices as well as wavelet-filtered features across multiple frequency bands (see Table 1). Radiomic features can be broadly categorized into three main classes: first-order, shape, and texture features. First-order (intensity-based) features describe the distribution of voxel intensities within the region of interest (ROI), independent of spatial relationships. These include metrics such as mean, variance, entropy, energy, uniformity, percentiles, mean absolute deviation (MAD), root mean square error (RMSE), kurtosis, and skewness. Shape features characterize the geometry and morphology of the segmented region, including volume, surface area, sphericity, and compactness. Texture features capture intra-lesional heterogeneity by quantifying spatial relationships among voxels and are derived from several statistical matrices: (i) the Gray Level Co-occurrence Matrix (GLCM), which describes co-occurrences of gray levels at fixed distances and angles (e.g., contrast, correlation, homogeneity, joint entropy/energy, autocorrelation, cluster tendency/shade/prominence, inverse variance, MCC); (ii) the Gray Level Run Length Matrix (GLRLM), which encodes consecutive voxels with the same gray level (e.g., short-/long-run emphasis, high-/low-gray-level run emphasis, run entropy, run variance, run percentage, and non-uniformity); (iii) the Gray Level Size Zone Matrix (GLSZM), which measures connected areas of equal intensity (e.g., small/large area emphasis, high-/low-gray-level zone emphasis, zone entropy/variance, zone percentage, and non-uniformity); (iv) the Gray Level Dependence Matrix (GLDM), which quantifies the degree of dependence among neighboring voxels (e.g., small/large dependence emphasis, high-/low-gray-level emphasis, dependence variance/entropy, gray-level variance, and non-uniformity); and (v) the Neighborhood Gray Tone Difference Matrix (NGTDM), which evaluates local intensity differences with respect to neighboring voxels (e.g., coarseness, contrast, busyness, complexity, and strength).

To ensure comparability and reproducibility between the two platforms, identical extraction settings were applied. This included selecting all feature families, maintaining default discretization settings, and activating wavelet filtering with default parameters (decomposition level 1 and Coiflet 1 mother wavelet) (Table 1).

### 2.4. Statistical Analysis

An initial evaluation of intra-software radiomic feature robustness was performed to assess the potential variability introduced by user interaction during the semi-automatic segmentation process. For a representative subset of patients, three independent segmentations of each lesion were generated using the same semi-automatic algorithm but with slightly different manual initializations provided by the operator. This approach enabled the quantification of feature stability and repeatability under small variations in the manual input that initializes the segmentation algorithm.

The Intraclass Correlation Coefficient (ICC) was used to assess the reliability of radiomic features across repeated measurements. Specifically, the ICC(A,1) model was adopted, which corresponds to a two-way mixed-effects model for single measurements under the criterion of absolute agreement [22]. This formulation considers both random error and systematic differences between measurements, providing a comprehensive estimate of reproducibility. The ICC(A,1) is defined as:

(1) $$ICC(A,1)=\frac{MS_{R}-MS_{E}}{MS_{R}+(k-1)MS_{E}+\frac{k(MS_{C}-MS_{E})}{n}}$$

where $MS_{R}\;$is the mean square between subjects, $MS_{C}\;$is the mean square between measurements, $MS_{E}\;$is the residual mean square error, k is the number of repeated measurements, and n is the number of subjects. Interpretation of ICC values followed established thresholds [23]: values <0.50 indicate poor reliability, 0.50–0.75 moderate reliability, 0.75–0.90 good reliability, and >0.90 excellent reliability.

In this study, only features with ICC(A,1) ≥ 0.75 were considered robust and retained for subsequent analyses. This filtering step was applied independently to the feature sets derived from each software platform, ensuring that only stable and reproducible features were used in subsequent cross-software robustness evaluations.

To quantify the similarity or dissimilarity between radiomic features, several distance metrics were computed for both datasets (Platform A and Platform B). These distance matrices served as the input for hierarchical clustering and directly influenced the resulting cluster structures. The following metrics were employed:

- Pearson Correlation Coefficient (PCC):

Measures the linear relationship between two variables. For clustering purposes, the correlation values were converted into an equivalent Euclidean distance according to:

(2) $$d_{\mathrm{corr}}(j,k)=\sqrt{2(1-r_{jk})}$$

where $r_{jk\;}$is the Pearson correlation coefficient between features j and k [24].

- Spearman Correlation:

A non-parametric measure of monotonic association based on feature ranks. Similarly to the Pearson approach, it was transformed into a distance metric as:

(3) $$d_{\mathrm{rank}}(j,k)=\sqrt{2(1-\rho_{jk})}$$

where $\rho_{jk}\;$is the Spearman rank correlation coefficient [24].

- Euclidean Distance (L2):

The standard straight-line distance between two points in a multi-dimensional feature space, defined as:

(4) $$d_{E}\left(j,k\right)=\sqrt{\sum_{i=1}^{N}(x_{ij}-x_{ik}{)}^{2}}$$

This metric is compatible with Ward’s linkage criterion, commonly used in hierarchical clustering [25].

- Manhattan Distance (L1):

Also known as *taxicab distance*, it represents the sum of the absolute differences between feature values:

(5) $$d_{M}(j,k)=\sum_{i=1}^{N}|x_{ij}-x_{ik}|$$

This measure is generally more robust to outliers than Euclidean distance [24].

- Cosine Distance:

Evaluates the orientation similarity between two feature vectors, independent of their magnitude. It is derived from the cosine similarity measure:

(6) $$d_{\cos}(j,k)=1-\frac{\sum_{i=1}^{N}x_{ij}x_{ik}}{\sqrt{\sum_{i=1}^{N}x_{ij}^{2}}\;\sqrt{\sum_{i=1}^{N}x_{ik}^{2}}}$$

where smaller values indicate higher similarity [25].

Agglomerative hierarchical clustering was applied to group redundant radiomic features by iteratively merging the most similar pairs of observations, thereby constructing a dendrogram that represents the hierarchical structure of feature relationships. This method is particularly advantageous for identifying highly correlated feature groups and reducing data dimensionality without requiring a predefined number of clusters (k) [26,27].

Several linkage methods were tested to evaluate their influence on cluster formation [25,28,29,30,31,32]:

- Ward.D2: Minimizes the total within-cluster variance at each merging step, requiring the use of Euclidean distances.
- Average linkage (UPGMA): Computes the average distance between all pairs of observations belonging to two clusters.
- Complete linkage: Considers the maximum pairwise distance between observations in two clusters, promoting the formation of compact, evenly sized clusters.
- Single linkage: Uses the minimum distance between observations in different clusters, which can lead to the chaining effect in the presence of noise or outliers.

The optimal number of clusters (k) was determined using the NbClust package in R, which evaluates multiple internal validation indices (e.g., Silhouette, Dunn, and Calinski–Harabasz) and proposes a consensus solution based on the majority rule [33,34]. An early-stop mechanism was implemented to limit computational complexity: if the majority support for a given number of clusters consistently fell below a predefined threshold for several consecutive iterations, the search was automatically halted.

To assess cross-platform clustering stability, the Adjusted Rand Index (ARI) was computed between the partitions obtained from Platform A and Platform B for each combination of distance metric and linkage method [35,36,37]. The ARI quantifies the agreement between two clustering solutions while correcting for chance, with values near 0 indicating random agreement and values approaching 1 indicating perfect concordance. A stability criterion of ARI ≥ 0.8 was adopted to identify robust clustering configurations [36]. When multiple configurations met this threshold, the corresponding solutions were aggregated by consensus to ensure consistent feature grouping across platforms.

To quantify the cross-tool robustness of individual radiomic features between Platform A and Platform B, a Composite Index (CI) was computed [38]. This index integrates three complementary dimensions that capture different aspects of feature consistency:

1. Shape Consistency: measured by the Pearson correlation coefficient ($r_{f}$) between feature values extracted from the two platforms.
2. Distributional Similarity: assessed using the two-sample Kolmogorov–Smirnov (KS) statistic ($D_{f}$), which evaluates whether the two feature distributions differ significantly.
3. Absence of Scale Bias: quantified through the Mean Fractional Ratio (MFR), a stabilized log-ratio comparing the mean feature values across platforms.

All metrics were standardized using a robust z-score transformation, and the final Composite Index for each feature f was defined as:

$$CI_{f}=z_{\mathrm{corr},f}-z_{\mathrm{KS},f}-z_{\mathrm{MFR},f}$$

where $z_{\mathrm{corr},f}$ is the z-value of the correlation coefficient for feature f, $z_{\mathrm{KS},f}$ is the z-value of the Kolmogorov–Smirnov test statistic for feature f and $z_{\mathrm{MFR},f}$ is the z-value of the MFRs for feature f.

Consistent sign conventions were applied so that higher $CI_{f\;}$values correspond to strong correlation, low distributional difference, and minimal scale bias. Thus, a high CI value indicates greater cross-platform robustness of the feature.

Features with $CI_{f}\;$exceeding a predefined stability threshold were designated as cluster “winners”, representing the most robust feature within each cluster. To account for features with comparable performance, a “near-winner” strategy was also implemented, including those with CI values close to the cluster winner. Therefore, “Winners” were defined as the highest-ranked feature within each cluster by the Composite Index (CI), which integrates inter-platform correlation, distributional similarity (KS), and absence of scale bias (mean fractional ratio). “Near-winners” had CI values comparable to the cluster leader.

Additional quality control filters were then applied, including (i) a minimum acceptable *A*–*B* Pearson correlation and (ii) an intra-cluster redundancy filter to prevent highly correlated features from being retained simultaneously. The outcome of this multi-step selection process was a final subset of robust, non-redundant, and cross-validated radiomic features, suitable for downstream predictive modeling and cross-platform comparative analyses.

All statistical analyses were conducted using R (RStudio, version 4.4.2).

**Figure 2:**
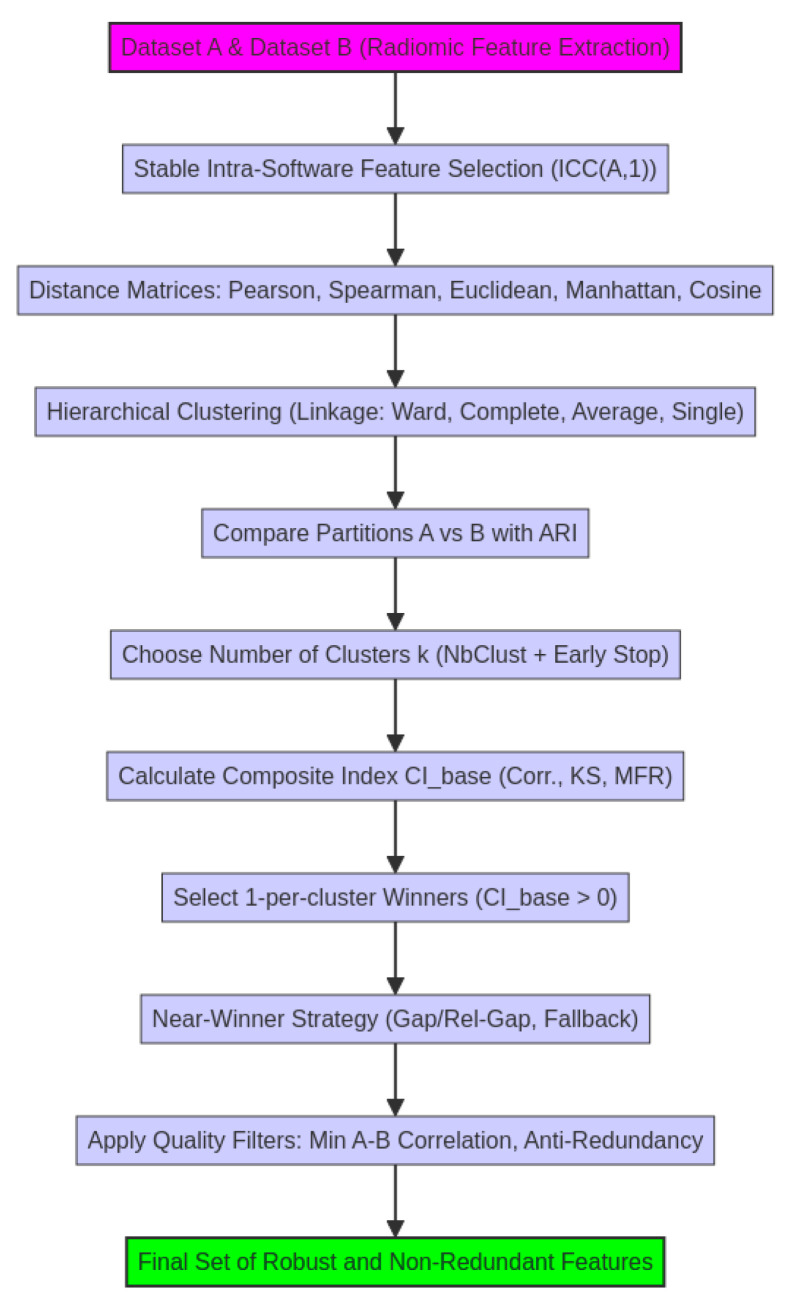
Workflow for cross-software radiomic feature robustness analysis.

Figure 2 reports the workflow of the pipeline.

## 3. Results

The intra-software robustness analysis, based on the ICC(A,1) model, demonstrated that a substantial proportion (≥95%) of radiomic features exhibit good to excellent reliability when extracted under slightly varying segmentation conditions by the same operator.

The results indicated that k = 2, 3, and 5 were the most frequently suggested cluster numbers across multiple internal indices (e.g., Silhouette, Dunn, Calinski–Harabasz), reflecting some variability in the underlying feature space structure, from more complex and articulated organizations (k = 5) to more compact, binary ones (k = 2).

Cross-software stability was then evaluated by comparing the clustering partitions obtained from Platform A and Platform B using the Adjusted Rand Index (ARI). Several combinations of distance metrics and linkage methods yielded high ARI values, with multiple configurations reaching ARI = 1.0, indicating perfect concordance between the two software platforms. Notably, setups involving Pearson or Spearman correlation distances combined with single or centroid linkage frequently achieved this perfect agreement. Nevertheless, internal clustering metrics such as the Silhouette and Dunn indices revealed variations in cluster compactness and separability, indicating that not all high-ARI configurations corresponded to equally stable cluster structures.

Among the top-performing solutions based on ARI, emphasis was placed on configurations that also demonstrated satisfactory internal validity. The most promising setups for feature selection typically involved Pearson distance combined with centroid or single linkage.

**Figure 3:**
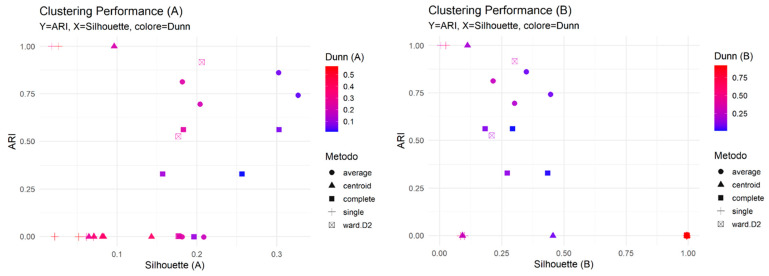
Clustering performance evaluation for Dataset A and Dataset B. The scatter plots illustrate the performance of hierarchical clustering across various linkage methods (indicated by shape) for (**A**) Dataset A and (**B**) Dataset B. The y-axis represents the Adjusted Rand Index (ARI), which measures the stability and consistency of the clustering partitions. The x-axis represents the Silhouette Score, an internal validation metric for cluster compactness and separation. The color gradient of the points indicates the Dunn Index (color = Dunn Index), another internal metric evaluating the ratio of minimum inter-cluster distance to maximum intra-cluster distance. Shape indicates linkage method (average, centroid, complete, single, ward.D2).

Figure 3 presents the multi-metric evaluation of clustering performance and stability across Dataset A and Dataset B. The plots are organized to simultaneously assess stability (Adjusted Rand Index, ARI, on the y-axis) and internal quality (Silhouette Score on the x-axis and Dunn Index via color gradient) for various distance and linkage method combinations. A high ARI value (near 1.0) was prioritized, indicating a highly consistent clustering partition across the two datasets. We observed that certain linkage methods, such as centroid and ward.D2, consistently yielded the highest ARI scores, particularly in Dataset B, suggesting they produce the most stable partitions. Importantly, the analysis revealed a trade-off: the most stable solutions (highest ARI) often exhibited moderate or low internal quality metrics (Silhouette and Dunn Indices). This finding highlights the necessity of prioritizing inter-dataset stability (ARI) over internal compactness when selecting the optimal clustering method for robust feature selection.

Panel A (Dataset A). ARI and Silhouette display a positive association; most solutions have modest Silhouette (≈0.05–0.30) with intermediate–high ARI (≈0.6–1.0), indicating good agreement with the external labels despite moderate compactness. Panel B (Dataset B). The same pattern holds, with several solutions achieving higher Silhouette (≈0.20–0.55) alongside high ARI (≈0.7–1.0). Notably, a single-linkage solution shows Silhouette ≈ 1.0 but ARI ≈ 0, i.e., a degenerate partition that is internally very compact yet inconsistent with the external reference. In contrast, ward.D2 and complete provide the best compromise (concurrently high ARI, reasonable Silhouette, and higher Dunn).

Within each selected clustering solution, “winner” features defined as the most representative feature in each cluster according to the Composite Index (CI_f) were identified.

The listed features (Table 2) appeared as cluster winners in the indicated number of solutions, highlighting the predominance of wavelet-transformed descriptors—particularly those derived from GLCM cluster shade and first-order statistics (e.g., mean)-as the most robust radiomic features across software platforms.

Additionally, “near-winner” features, defined as those with CI values comparable to their cluster winner, were also identified. The frequency analysis of both winner and near-winner features across all analyzed solutions revealed that certain features, such as original_firstorder_skewness, wavelet_lhl_firstorder_mean, and wavelet_hlh_glcm_clustershade, appeared consistently across nearly all solutions (Table 3).

Across clustering solutions validated by the Adjusted Rand Index, the most frequently selected winner features were predominantly wavelet-transformed first-order and GLCM descriptors, with recurrent appearances of firstorder_skewness/mean/median in specific wavelet sub-bands and GLCM statistics such as cluster shade and IMC2. Additional, but less frequent, winners arose from GLSZM (zone percentage), GLDM (dependence non-uniformity normalized), and NGTDM (strength). These families differ in both the underlying image property and spatial scale they quantify: first-order moments summarize the voxel-intensity distribution within the ROI; GLCM winners reflect direction- and distance-dependent second-order relationships (e.g., asymmetry and redundancy of gray-level co-occurrences); GLSZM zone percentage encodes the proportion of small homogeneous zones; GLDM dependence non-uniformity normalized captures variability in local voxel dependencies after normalization; and NGTDM strength measures neighborhood contrast relative to local means. Notably, winners were enriched in wavelet sub-bands combining low/high filtering along different axes (e.g., LHL, HLH, HHL), indicating that intermediate spatial frequencies and orientation-specific patterns contributed most to cross-platform stability. Consistent with this, winner features showed high inter-platform concordance (strong rank/linear correlations), low distributional divergence (small KS distances), and a mean fractional ratio close to unity, whereas near-winners exhibited comparable but slightly inferior Composite Index values. Taken together, these patterns suggest that (i) scale-selective pre-filtering stabilizes first-order and co-occurrence statistics against platform-specific gray-level handling, and (ii) ratio-/normalization-based formulations (e.g., IMC2, zone percentage, normalized non-uniformity) further enhance reproducibility.

## 4. Discussion

This study proposed and validated a comprehensive and reproducible workflow to assess the robustness of radiomic features both within and across software platforms. The results demonstrate that the combined use of intra-software reliability testing, cross-software clustering validation, and composite feature evaluation enables the identification of a consistent subset of stable, reproducible, and non-redundant radiomic descriptors. Such methodological rigor is essential to ensure the translational potential of radiomic biomarkers, a challenge emphasized by recent consensus guidelines and reproducibility studies [16,39,40].

The intra-software analysis, based on the ICC(A,1) model, confirmed that most features achieved good-to-excellent reliability under small variations in segmentation, aligning with prior observations that semi-automatic delineation remains a critical source of variability [5,23,39]. The use of an ICC ≥ 0.75 threshold, consistent with established radiomic standards [16], proved effective in excluding unstable descriptors and preserving a reliable core set for subsequent analyses.

The cross-software robustness assessment represented the most innovative component of the workflow. By integrating multiple distance metrics and linkage methods within a hierarchical clustering framework, and by validating inter-software agreement through the Adjusted Rand Index (ARI), the study demonstrated that feature groupings derived from the MM Radiomics of syngo.via Frontier and the PyRadiomics module by 3D Slicer can achieve high structural concordance (ARI ≈ 1.0). This finding supports the feasibility of harmonizing radiomic analyses across distinct IBSI-compliant environments [16,22,24].

Our results align with previous methodological studies emphasizing the importance of repeatability and standardization in radiomics [41,42,43,44,45,46,47,48,49,50,51,52,53,54]. Jha et al. demonstrated that only a subset of features retains high test–retest reproducibility across scanners and acquisition settings [37], while Escudero Sánchez et al. highlighted the influence of CT slice thickness on feature stability [42]. Similarly, Gong et al. recently showed that selecting only highly repeatable features enhances the cross-institutional generalizability of prognostic models [46]. Together, these findings support the rationale of the current study, namely, that robustness assessment and redundancy reduction are prerequisites for reliable predictive modeling.

The Composite Index (CI), combining correlation, Kolmogorov–Smirnov distributional similarity, and mean fractional ratio, provided a multidimensional measure of cross-software consistency, extending beyond conventional correlation-based analyses. This holistic quantification echoes the methodological philosophy of Qiu et al. [44], who used hierarchical clustering to identify reproducible and non-redundant features, and of Oh et al. [45], who proposed a network-based framework for identifying robust radiomic clusters. However, the present study advances these approaches by explicitly validating inter-software stability through ARI and quantifying cross-platform reproducibility through CI, thus bridging structural and statistical robustness dimensions.

Across all clustering configurations, the most stable features were predominantly wavelet-transformed descriptors and first-order statistics, particularly cluster shade (GLCM-based) and mean values. The recurrent presence of original_firstorder_skewness, wavelet_lhl_firstorder_mean, and wavelet_hlh_glcm_clustershade across nearly all clustering solutions highlights their reproducibility and potential as cross-software imaging biomarkers. These results align with Hosseini et al. [43], who demonstrated that models based on robust features achieve improved predictive stability even if performance metrics (e.g., AUC) remain comparable to conventional models.

These findings are consistent with previous reports that wavelet features effectively capture tumor heterogeneity while maintaining relative insensitivity to acquisition and reconstruction differences [5,20,36].

Overall, the proposed workflow addresses key challenges in reproducibility and cross-platform variability, two of the major obstacles in radiomics standardization [16,40]. By combining intra- and inter-software validation with redundancy reduction, the framework ensures that only the most reliable and informative features are retained, thus enhancing both interpretability and predictive performance.

While our findings are directionally consistent with prior reports, this work advances radiomics toward clinical reliability by directly targeting a key translational bottleneck: software-induced variability. Using two widely adopted, IBSI-compliant platforms and identical 3D masks, we implement a stability-first selection framework that integrates (i) inter-platform concordance, (ii) distributional similarity, and (iii) scale-bias checks into a single Composite Index validated via clustering agreement. This framework yields a compact, interpretable shortlist of platform-agnostic, wavelet-enhanced first-order and texture descriptors that remained stable under routine CT conditions (portal venous phase, narrow slice thickness). Importantly, the analysis spans multiple hepatic/colorectal disease contexts at the lesion level, aligning the robustness criteria with how imaging biomarkers are deployed in practice.

By formalizing robustness as a pre-requisite gate, before model building, we provide a reproducible template for multi-center studies: (1) pre-register a cross-platform stability screen; (2) lock acquisition parameters and masks; (3) report per-feature “stability cards” (metric triplet + wavelet sub-band); and (4) propagate only stable features into downstream modeling. This stability-first pipeline and the resulting feature shortlist can seed harmonized, prospective validations, facilitate vendor-agnostic biomarker panels, and guide future work on scanner harmonization, segmentation variability, and outcome-linked thresholds.

Despite its strengths, this study has limitations. The dataset, although multi-cancer, included a relatively small sample size, which may limit the statistical power and generalizability of the findings. Moreover, the analysis was restricted to CT-based features extracted from colorectal cancer (CRC), liver metastases, and hepatocellular carcinoma (HCC); therefore, validation in larger, multi-center, and multi-modality cohorts is warranted.

Future studies should validate this approach on larger, multi-center datasets and expand it to other imaging modalities, ultimately fostering reproducible and generalizable quantitative imaging biomarkers for precision oncology.

## 5. Conclusions

This study introduced and validated a robust, multi-stage framework for evaluating radiomic feature stability across software platforms. By combining intra-software reliability assessment, hierarchical clustering with ARI-based validation, and a Composite Index-driven feature selection strategy, the proposed workflow identified a compact set of reproducible, non-redundant, and transferable radiomic features.

Most of these stable features were wavelet-derived or first-order statistics, confirming their reliability across IBSI-compliant platforms. Their consistent behavior highlights their potential to serve as robust imaging biomarkers for predictive modeling.

The framework offers a reproducible methodology for harmonizing radiomic analyses, contributing to the broader goal of cross-software standardization and clinical translation of radiomics.

## Figures and Tables

**Table 1:** Number of features per family in the two conditions (Platform A and Platform B).

Family	Platform A	Platform B
First-order	18	18
GLCM	24	24
GLDM	14	14
GLRLM	16	16
GLSZM	16	16
NGTDM	5	5
Shape	17	14
Wavelet	744	744
Total	854	851

**Table 2:** Most frequently selected “winner” features across clustering solutions. Within each optimal clustering configuration, a single “winner” feature per cluster was identified based on the highest Composite Index (CI_f) value, representing the most stable and informative descriptor in that cluster.

Feature	Number of Solutions
original_firstorder_skewness	27
wavelet_lhl_firstorder_mean	27
wavelet_hlh_firstorder_median	27
wavelet_lhh_firstorder_mean	27
wavelet_lhh_firstorder_skewness	27
wavelet_hll_firstorder_median	27
wavelet_hhl_glcm_clustershade	27
wavelet_lhl_firstorder_median	27
wavelet_lhl_glcm_clustershade	27
wavelet_hlh_glcm_clustershade	27
wavelet_hhh_glcm_imc2	16
wavelet_hlh_firstorder_mean	15
wavelet_hhl_glszm_zonepercentage	14
wavelet_hhl_ngtdm_strength	14
wavelet_lhh_glszm_zonepercentage	11
wavelet_hhh_gldm_dependencenonuniformitynormalized	10
wavelet_hhl_gldm_dependencenonuniformitynormalized	10
wavelet_hll_glcm_jointenergy	9
wavelet_lhl_gldm_dependencenonuniformitynormalized	8
wavelet_hlh_glcm_correlation	8

**Table 3:** Frequency of “winner” and “near-winner” features across all evaluated clustering solutions. This table reports the number of solutions in which each feature was identified either as a “winner” or as a “near-winner,” i.e., features with Composite Index values comparable to the cluster winner.

Feature	Number of Winning Solutions
original_firstorder_skewness	22
wavelet_lhl_firstorder_mean	21
wavelet_hlh_firstorder_median	20
wavelet_lhh_firstorder_mean	20
wavelet_lhh_firstorder_skewness	19
wavelet_hhl_glcm_clustershade	18
wavelet_hlh_glcm_clustershade	18
wavelet_hhh_glcm_imc2	15
wavelet_lhl_firstorder_median	14
wavelet_hll_firstorder_median	12
wavelet_hhl_glszm_zonepercentage	11
wavelet_hhl_ngtdm_strength	10
wavelet_lhh_glszm_zonepercentage	8
wavelet_hhh_gldm_dependencenonuniformitynormalized	7
wavelet_hhl_gldm_dependencenonuniformitynormalized	7
wavelet_hll_glcm_jointenergy	6
wavelet_lhl_gldm_dependencenonuniformitynormalized	6
wavelet_hlh_glcm_correlation	5

## Data Availability

The original contributions presented in this study are included in the article and at link https://zenodo.org/records/17643762.
